# Calculation Method for Phenotypic Traits Based on the 3D Reconstruction of Maize Canopies

**DOI:** 10.3390/s19051201

**Published:** 2019-03-08

**Authors:** Xiaodan Ma, Kexin Zhu, Haiou Guan, Jiarui Feng, Song Yu, Gang Liu

**Affiliations:** 1 College of electrical and information, Heilongjiang Bayi Agricultural University, Daqing 163319, China; mxd@cau.edu.cn (X.M.); zhukexin0319@163.com (K.Z.); JRFeng_bynd@163.com (J.F.); 2 Agronomy College of Heilongjiang Bayi Agricultural University, Daqing 163319, China; byndys@163.com; 3 Key Laboratory of Modern Precision Agriculture System Integration Research, Ministry of Education, China Agricultural University, Beijing 100083, China

**Keywords:** maize, 3D point cloud, pre-processing, phenotypic traits, calculation method

## Abstract

A reasonable plant type is an essential factor for improving canopy structure, ensuring a reasonable expansion of the leaf area index and obtaining a high-quality spatial distribution of light. It is of great significance in promoting effective selection of the ecological breeding index and production practices for maize. In this study, a method for calculating the phenotypic traits of the maize canopy in three-dimensional (3D) space was proposed, focusing on the problems existing in traditional measurement methods in maize morphological structure research, such as their complex procedures and relatively large error margins. Specifically, the whole maize plant was first scanned with a FastSCAN hand-held scanner to obtain 3D point cloud data for maize. Subsequently, the raw point clouds were simplified by the grid method, and the effect of noise on the quality of the point clouds in maize canopies was further denoised by bilateral filtering. In the last step, the 3D structure of the maize canopy was reconstructed. In accordance with the 3D reconstruction of the maize canopy, the phenotypic traits of the maize canopy, such as plant height, stem diameter and canopy breadth, were calculated by means of a fitting sphere and a fitting cylinder. Thereafter, multiple regression analysis was carried out, focusing on the calculated data and the actual measured data to verify the accuracy of the calculation method proposed in this study. The corresponding results showed that the calculated values of plant height, stem diameter and plant width based on 3D scanning were highly correlated with the actual measured data, and the determinant coefficients R^2^ were 0.9807, 0.8907 and 0.9562, respectively. In summary, the method proposed in this study can accurately measure the phenotypic traits of maize. Significantly, these research findings provide technical support for further research on the phenotypic traits of other crops and on variety breeding.

## 1. Introduction

The selection and breeding of excellent maize varieties has attracted extensive international attention [1]. Many influential research centers, such as the International Maize and Wheat Improvement Center (CIMMYT) [2] and the International Center for Agricultural Research in the Dry Areas (ICARDA) [3] have been working on genetic diversity and cultivation of maize plants. Maize is also one of the most important food crops in China, as well as one of the most important industrial raw materials and economic crops. Deep processing of maize can be widely applied in food, medicine, bioenergy and more than 20 other industries. The development of the Chinese maize industry is inseparable from maize breeding. However, with the increases in drought, diseases and insect pests, the resistance of new maize varieties must be improved further [4]. Thus, maize breeding research faces a series of challenges [5].

The crop phenotype refers to the physical, physiological and biochemical characteristics and properties of crops that are determined or influenced by genetic and environmental factors during their growth and development [6,7,8]. Accurate and rapid acquisition of plant phenotypic information will provide theoretical and technical support for promoting the development of crop science, thus ensuring world food security, ecological security and sustainable agricultural development.

Plant height is a vital phenotypic trait for characterizing plant growth and is one of the most basic indicators in plant morphological investigations. Plant height is defined as the distance from the base of the plant to the upper boundary of the main photosynthetic tissues (excluding inflorescences) [9,10]. The maintenance of plant height at a relatively uniform horizontal height in the field indicates a well-distributed nutrient supply of fertilizer in the field, which is conducive to processes such as photosynthesis and pollination. Stem diameter is another important trait reflecting plant phenotype [11,12]. In addition to its roles in water and nutrient transport, the stem can store nutrients and can transport nutrients to grains in later stages. Furthermore, the stem is the organ that produces and supports spikes, exhibiting phototropism and negative geotropism [13]. When the plant lodges, it can bend upward to maintain growth, so that the plant can become erect again to reduce losses. The thickness of the stem is positively correlated with lodging resistance [10], as well as the ability to store and transport water and fertilizer, resulting in a greater amount of nutrients being transported to the grain. Additionally, canopy breadth is the average width of the plant canopy, which not only determines the distribution of light in the plant canopy but also serves as an index for evaluating the efficiency and effectiveness of agricultural management in relation to fertilization, irrigation, thinning and harvesting [14,15]. Therefore, monitoring the changes in plant height, stem diameter and canopy breadth in different periods can enable agronomists and breeders to keep abreast of plant health and growth in a timely manner.

With the development of agricultural mechanization, as well as modern farmland cultivation and management technology, significant improvements in agricultural production technology have been achieved [16]. Revolutionary changes in maize science research have been obtained during the development of modern biotechnology [17]. However, traditional methods for the acquisition of phenotypic traits, such as plant height, stem diameter and canopy width, are still carried out through manual measurement with a ruler or measuring tape, which is labor-intensive, inefficient and inaccurate and limits the development of modern maize science [18,19]. In view of the current status, it is urgent to develop non-destructive and accurate means of detection of phenotypic traits to reduce labor, improve efficiency and promote the rapid development of maize science [20,21]. In this context, informatization has been widely applied to the study of maize phenotypic traits [22].

One important means of studying the calculation methods for maize canopy phenotypic parameters is based on reconstruction of the three-dimensional (3D) structure of the maize canopy [23]. At present, the methods for 3D maize canopy acquisition mainly include the use of 3D digitizers, hand-held sensors, binocular stereo vision technology, laser scanning technology and unmanned aerial vehicle (UAV) remote-sensing technology [24]. It is time-consuming to manually measure a large amount of geometric information from crop canopies when reconstructing 3D plant morphology with a 3D digitizer [25]. Hand-held sensors use a contact method to measure geometric parameters, which leads to deformation of the plant canopy [26]. Furthermore, visible light-based binocular stereo vision technology possesses advantages in the non-contact and non-destructive acquisition of crop canopy images without destroying crop growth patterns, resulting in significant improvement of the efficiency of data acquisition [27], which is helpful for monitoring crop emergence rates, flowering dynamics, canopy coverage and lodging during crop growth [28]. However, the quality of images acquired by this technique is greatly affected by light conditions; hence, the accuracy of the calculation of phenotypic parameters needs to be further improved. Laser [29] or light detection and ranging (LIDAR) [15] sensors are active remote-sensing devices using laser as the emitting light source and photoelectric detection. These devices present the advantages of a high resolution, strong anti-jamming ability and good detection performance at low altitudes, and can quickly obtain high-precision horizontal and vertical structure parameters of plant canopies. However, due to their high cost and the massive data processing involved, these devices are generally only applied in the analysis of tree biomass [30] and are rarely used in crop phenotypic research. In addition, the analysis of crop phenotypic traits based on UAV multi-sensor platforms presents obvious superiority, due to factors such as a low cost, high efficiency, and high-resolution data acquisition, as well as synchronous acquisition of multi-source images [31]. This strategy has been widely used in analyzing parameters such as plant height, chlorophyll content, and nitrogen content [32]. However, it is difficult to measure other important phenotypic traits of crops, such as the leaf inclination and stem diameter of maize, with UAV remote-sensing technology. High-throughput phenotyping platforms in greenhouses with controlled growth conditions are alternative approaches for automatically measuring geometric dimensions of plants [33,34]. Hyperspectral imaging technology [35], two dimensional digital camera [36] and Structure from Motion (SfM) method [37] contribute significantly to the automatic phenotyping greenhouses in the aspects of morphological characteristics [38] and stress resistance [39]. Although these platforms can be operated automatically with good repeatability, they also show limitations. The main shortcoming is that certain traits calculated from an individual plant can not reflect the true cases in the natural environment due to the artificial laboratory conditions.

Although these excellent sensors have been widely used in the high-throughput acquisition of plant phenotypic parameters, there are still some limitations in obtaining specific phenotypic traits. In addition, advanced improvement is warranted concerning the accuracy of plant 3D reconstruction and the calculation accuracy of phenotypic traits.

Hand-held laser scanning, which shows benefits in the high-precision acquisition of 3D point clouds, plays a crucial role in plant 3D reconstruction and the calculation of plant phenotypic traits, especially in acquiring more specific phenotypic traits of crops [40] (e.g., stem diameter, leaf inclination angle, blade angle). The hand-held scanner can digitize the surface of any object rapidly and conveniently and displays the morphological structure of the target object in 3D space, providing an effective data source for further calculation of phenotypic traits.

In this context, to compensate for the shortcomings of the research results above and improve the calculation accuracy for phenotypic traits, a 3D structural model of maize plants was established through FastSCAN hand-held laser scanning (Cobra™, Aranz Scanning Ltd., New Zealand, Christchurch). On this basis, the calculation method for maize phenotypic traits was subsequently studied. The main aims of this study were as follows: (1) to simplify raw point clouds using the grid method; (2) to progressively remove the effect of noise on point cloud quality in the maize canopy with a bilateral filtering algorithm; and (3) to calculate phenotypic traits of the maize canopy, including plant height, stem diameter and canopy breadth by fitting spheres and cylinders based on 3D reconstruction of the maize canopy. The results of this research may provide technical support for further exploration of the phenotypic traits of other crops and the breeding of crop varieties.

## 2. Materials and Methods

### 2.1. Experimental Treatments and Measurement of Phenotypic Traits

From May to September 2018, maize planting and data acquisition were carried out in the Innovation and Entrepreneurship Training Park for Excellent Agricultural Talents of the Agronomy College of Heilongjiang Bayi Agricultural University (46°62′ N, 125°20′ E), which is located in the north temperate zone, with a continental monsoon climate. The average temperature is 4.2 °C, the annual average frost-free period is 143 days, annual rainfall is 427.5 mm, and annual evaporation is 1635 mm. The effective accumulated temperature is 2600 °C, and there are 1147.8 sunshine hours.

The pot cultivation method was adopted in the experiment. Each pot (Polyvinyl chloridematerial, 32 cm in diameter and 26 cm in height) was filled with 10 kg of soil (dried soil). Natural dried and full maize seeds with a consistent size were selected. The two varieties of maize seeds were soaked and disinfected with 1% NaClO for 5 min and then rinsed with distilled water 3 times. Finally, the sterilized seeds were placed in a germination box lined with two layers of wet filter paper and then placed in a 25 °C constant temperature box for one day. The soil tested was meadow soil, and maize was planted at 10-day intervals in three batches. Each variety was repeated five times and placed randomly. Each pot was sown evenly, and the seedlings were transplanted individually at the trefoil stage after emergence. The plants were watered to 70 percent of the soil moisture content each time; other aspects were in accordance with regular management practices. The study of phenotypic traits from the trefoil stage to the jointing stage is particularly important among the total growth stages of maize. Accordingly, phenotypic traits including plant height, stem diameter and canopy breadth were measured with rulers and measuring tapes from the trefoil stage to the jointing stage, to verify the validity of the method for calculating phenotypic traits.

### 2.2. Data Acquisition Device

To reconstruct the precise 3D structure of the maize canopy and improve the accuracy of point cloud data in the maize canopy, emphasis should be placed on the improvement of data acquisition technology for the maize canopy. In this study, a FastSCAN Cobra™ hand-held 3D laser scanner was used to acquire 3D point cloud data for the maize canopy. The FastSCAN device can scan non-metallic and opaque objects. Based on the operational principle, the scanner records data on a contour section of an object surface by projecting a laser beam. The embedded motion tracking technology is applied to the position and orientation of the detection handle and to reconstruct the object in three dimensions jointly. During the process, the resolution of the device is 0.178 mm in the range of 200 mm from the scanned object, and the scanning speed is 50 lines per second. The distance between lines depends on the moving speed of the laser head, and the resolution is 1 mm at the moving speed of 50 mm per second. The speed at which the wand is moved over the surface of the object is the major determinate of the resolution of the sweeps. The number of raw 3D cloud points increased with the growth of maize from over 10,000 points in the 3-leaves period to over 40,000 points in the 8-leaves period.

**Figure 1:**
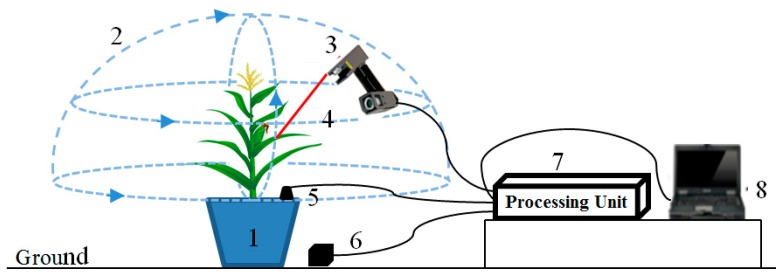
Schematic diagram of the data acquisition process. (1) Maize plant; (2) Scan range; (3) Wand; (4) Laser scanning line; (5) The receiver; (6) The transmitter; (7) Processing unit; (8) Laptop.

Figure 1 is a schematic diagram of the data acquisition process. The scanning effect can be viewed in real time with FastSCAN software installed in a laptop. To ensure both a high resolution and high accuracy, the wand should be held close to the maize plant’s surface during scanning, but no closer than 80 mm to remain within the camera’s field of view. Parts of the surface closer to receiver will be scanned more accurately. The transmitter should be kept close to the wand and receiver, as accuracy deteriorates with distance, but no closer than 100 mm to avoid signal overload. The maximum separation of any two components is approximately 750 mm, but they should be kept as close as possible for best results.

### 2.3. Overall Process Flow for Calculating Phenotypic Traits

In this study, a method based on the use of a FastSCAN hand-held laser scanner was proposed to obtain a point cloud for a maize canopy and accurately reconstruct the 3D structure of maize plants. On this basis, the phenotypic traits of maize plants were calculated through the steps listed below.

**Figure 2:**
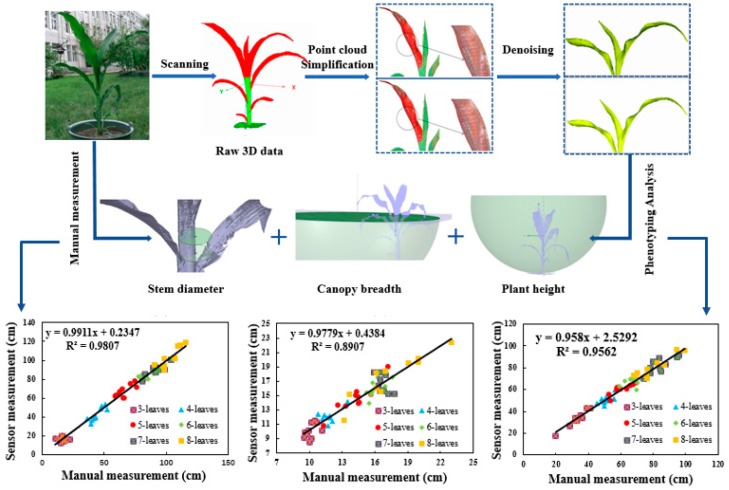
Flow chart for calculating the phenotypic traits of maize plants.

First, the original 3D point clouds of maize plants from the trefoil stage to the jointing stage were obtained with a FastSCAN hand-held laser scanner. Then, to avoid the interference of the large amount of data redundancy generated by the 3D scanner and subsequent space occupation, and to further improve the speed of data processing, a simplification approach for the 3D point cloud with adaptive density based on the grid method was used to simplify the raw 3D data of the maize plants. On this basis, bilateral filtering was applied to progressively denoise the maize canopy to reduce the impact of noise on the accuracy of the 3D reconstruction of the maize canopy. In addition, phenotypic traits including plant height, stem diameter and canopy breadth were calculated on the basis of accurate reconstruction of the 3D structure of maize plants. In the last step, the validity of the algorithm proposed here was verified through linear regression analysis of the calculated and measured phenotypic traits. Figure 2 shows the flow chart for calculating the phenotypic traits of maize plants.

### 2.4. Pre-Processing of the Raw 3D Point Cloud

Due to the influence of the device itself and the external environment, the scanner may produce redundant data and noise in the process of obtaining the 3D point cloud of maize plants. Consequently, to obtain an accurate 3D structure of a plant, it is necessary to simplify the raw 3D point cloud and remove noise in advance, to improve the data processing speed and model accuracy. In this study, based on the characteristics of the point cloud data collected from the maize canopy, a simplification approach for 3D point clouds with adaptive density based on the grid method and a bilateral filtering algorithm for maize plants were proposed, which laid a foundation for accurately reconstructing the 3D structure of the maize canopy and calculating related phenotypic traits.

#### 2.4.1. Simplification Algorithm for Raw Data

The simplification of a point cloud is indispensable in 3D point cloud preprocessing. Data redundancies are not conducive to subsequent accurate 3D reconstruction, making the calculation time-consuming and directly affecting the speed and accuracy of storage and data processing. In this regard, it is necessary to simplify the raw 3D point cloud data.

To preserve the feature information of the point cloud, an adaptive density reduction method for 3D point clouds based on the grid method was proposed to simplify the original point cloud of the maize plants. In the first step, after reading all points in the original point cloud model, a spatial data index was established based on 3D grid method for the 3D point cloud. Thereafter, a cuboid box was established with the three sides paralleling the three axes of the coordinate system. The grid was therefore constructed with the coordinate of any point in the original 3D point cloud as $p_{i}\left(x_{i},y_{i},z_{i}\right)$, and the side length was expressed as follows:

(1) $$\begin{array}{c} X_{box}=X_{max}-X_{min}\\ Y_{box}=Y_{max}-Y_{min}\\ Z_{box}=Z_{max}-Z_{min} \end{array}$$

where, $X_{box}$, $Y_{box}$ and $Z_{box}$ represents the maximum range of the point cloud $p_{i}\left(x_{i},y_{i},z_{i}\right)$ along the x, y and z axes, respectively. The effect of point cloud simplification is affected by the size of the bounding box, and the side length of the bounding box has to be increased accordingly if the raw point cloud data are simplified. On the contrary, the average density of the point cloud increases with a decrease in the side length of the bounding box. The side length of the grid is as follows:

(2) $$D=\sqrt[{3}]{\gamma /\left(X_{box}Y_{box}Z_{box}/N\right)}$$

where γ is the coefficient of proportionality and N is the number of original 3D point clouds. The adjustable side length of the grid is defined as follows:

(3) $$D^{\prime }=\beta D$$

where β refers to the factor of proportionality that may be available for the adjustment of the side length of the grid. By integrating Formulas (2) and (3), the following formula can be obtained:

(4) $$D^{\prime }=\beta \sqrt[{3}]{\gamma X_{box}Y_{box}Z_{box}/N}$$

Analysis of the differences in the curvature and density of the 3D point clouds in different parts of the maize plant organs revealed that the density of the 3D point clouds of maize stems was relatively uniform, with relatively smaller deviation in the curvature of point clouds. Compared with the stems, the 3D point cloud density of maize leaves was greater, which was associated with relatively greater curvature deviation of point clouds. Furthermore, the proportionality factor β was utilized to adjust the side length of the grille, where the latter factor directly affected the efficiency of reduction. Hence, it was necessary to determine the curvature of the point cloud in space to select the appropriate proportionality factor β.

Covariance analysis is a common principal component analysis method for estimating normal vector and curvature selection. The covariance matrix of the point set is as follows:

(5) $$C={\left[\overset{\underset{p_{il}-\bar{p}}{p_{ik}-\bar{p}}}{\vdots }\right]}^{T}\cdot \left[\overset{\underset{p_{il}-\bar{p}}{p_{ik}-\bar{p}}}{\vdots }\right]$$

where $\bar{p}=\sum p_{ik}/k$ is the centroid of $N_{k}\left(p_{i}\right)$, $p_{ik}\in N_{k}\left(p_{i}\right)$. The value λ(i=0,1,2) of matrix Cproper is a non-negative intrinsic value, and the corresponding three eigenvectors $v_{i}\left(i=0,1,2\right)$ form an orthogonal basis. Plane $\left(\bar{x}-\bar{p}\right)\cdot v_{0}=0$ that minimizes the distance from the surrounding point of p to the plane is referred to a tangent plane; $v_{o}$ is the normal vector of the local surface at point $p_{i}$; and eigenvalue $\lambda_{o}$ is the variation of local surfaces along normal vectors. The calculation showed that the curvature of $\sigma_{k}\left(p_{i}\right)$ was close to that of $p_{i}$, which could therefore be used as the curvature value at this point.

(6) $$\sigma_{k}^{i}=\sigma_{k}\left(p_{i}\right)=\lambda_{o}/\lambda_{o}+\lambda_{1}+\lambda_{2}$$

With the leaf point cloud set as $\Omega =\left\{P_{i}\left(x_{i,}y_{i},z_{i}\right)|1\leq i\leq n\right\}$, and sampling point set as $Q=\left\{q_{i}\left(x_{i,}y_{i},z_{i}\right)|1\leq i\leq n\right\}$, for any point $q_{i}\left(x,y,z\right)$ in the set of sampling points, the curvature threshold can be expressed as follows:

(7) $$D=\frac{\sum_{i=1}^{N}\sigma_{i}}{N}$$

With the number of point clouds in the bounding box set as K, and point set as $W=\left\{P_{i}\left(x_{i,}y_{i},z_{i}\right)|1\leq i\leq K\right\}$, for any point $p_{i}\left(x,y,z\right)$, if the curvature $\sigma_{i}$ of the point is calculated, then the mean curvature of the point cloud is obtained as follows:

(8) $$D_{i}=\frac{\sum_{i=1}^{N}\sigma_{i}}{K}$$

If $D_{i}\leq D$, the grid is a non-detail bounding box with a relatively low curvature requirement and low density. By contrast, if $D_{i}>D$, then the grid is a detail bounding box with a high requirement of curvature and high density. Therefore, the proportionality factor is adjusted to screen the grid size, and the redundant 3D point cloud is simplified from the maize point cloud data.

#### 2.4.2. Denoising Algorithm for Raw Data

For better removal of noise in the 3D reconstruction of the maize canopy, it was necessary to divide the feature region of the maize canopy. The average curvature of the 3D point cloud was used to divide the maize plant area, where the single area had less feature information, and the rich area possessed more feature information.

The average curvature of any point $p_{i}\left(x,y,z\right)$ in the 3D point cloud is as follows:

(9) $$\bar{D}=\frac{\sum_{i=1}^{N}\sigma_{i}}{N}$$

**Figure 3:**
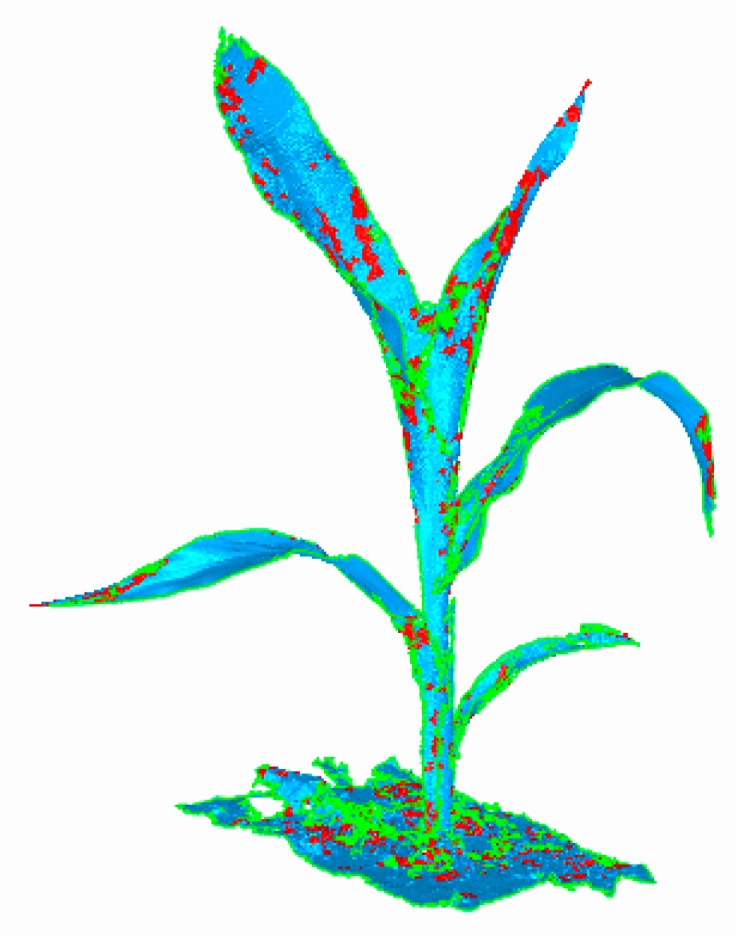
Characteristic region classification of three-dimensional (3D) point clouds of maize. Red points indicate the single region, other points represent rich region and are needed to be denoised by bilateral filtering algorithm.

To denoise different feature regions, the local eigenvalues at any point $p_{i}\left(x,y,z\right)$ in the 3D point cloud were compared with the threshold values. If the threshold was greater than the local eigenvalue, the point was marked as a single region (or conversely as a rich region). Additionally, different regions were divided for denoising, and the results of region division are shown in Figure 3. The maize plants were colorized with a gradient ramp of blue. The red parts of the plant represented the 3D cloud points with a single region, while the other parts, accounting for the majority of the maize plant, were used as rich regions, which had to be denoised further.

Subsequently, the 3D point clouds of maize with rich features were denoised with a bilateral filtering algorithm. In this study, the Jones bilateral filtering algorithm was applied to preserve the contour features of 3D point clouds in maize processing [41,42]. The algorithm is shown with Formula (10):

(10) $$p^{\prime }=p+\alpha \times n$$

where p represents the initial point cloud data; n is the normal vector; α is the bilateral filter coefficient; and $p^{\prime }$ refers to the point cloud data obtained after filtering. The calculation formula of α can be expressed as follows:

(11) $$\alpha =\frac{\sum_{i=1}^{k}w_{1}\left(\|p-p_{i}\|\right)w_{1}\left(\langle p-p_{i},n\rangle \langle p-p_{i},n\rangle \right)}{\sum_{i=1}^{k}w_{1}\left(\|p-p_{i}\|\right)w_{2}\left(\langle p-p_{i},n\rangle \right)}$$

where $w_{1}$ and $w_{2}$ represent the weights in the spatial and frequency domains of the bilateral filtering function, respectively, both of which control the smoothness and feature-preservation of bilateral filtering. Additionally, k is the sampling point in the nearest neighborhood of the sampling point. The concrete forms of $w_{1}$ and $w_{2}$ are shown with Formulas (12) and (13):

(12) $$w_{1}\left(x\right)=e^{-\frac{x^{2}}{2\sigma_{1}^{2}}}$$

(13) $$w_{2}\left(y\right)=e^{-\frac{x^{2}}{2\sigma_{2}^{2}}}$$

In the formulas, parameter $\sigma_{1}$ is the influence factor of the distance from point cloud data p to neighboring points on point p. The value of $\sigma_{1}$ is positively correlated with the number of neighborhood points, which is in positive proportion to the filtering effect, but inversely proportional to the ability of point cloud feature preservation. Furthermore, parameter $\sigma_{2}$ is the influence factor of the projection of the distance vector from data point p to the adjacent point on the normal n of the point in the point cloud data p. Parameter $\sigma_{2}$ regulates the degree of feature preservation of the point cloud data in the filtering process, which is proportional to the effect of feature preservation. Normally, $\sigma_{1}$ is the neighborhood radius of the point, and $\sigma_{2}$ is the standard deviation of the neighborhood point.

(14) $$\sigma_{1}=max\|p-p_{i}\|,i\in \left[1,k\right]$$

(15) $$\sigma_{2}=\sqrt{\frac{1}{k-1}\sum_{i=1}^{k}{\left(\xi_{i}-\bar{\xi }\right)}^{2}},x=\langle p-p_{i},n\rangle$$

The specific steps are as follows:

(1) Calculate the adjacent point k of each data point $p_{i}$ in the region of the 3D model rich in feature information;

(2) For each adjacent point k, calculate the value of $x=\|p-p_{i}\|$ in parameter $w_{1}\left(x\right)$ and the value of $y=\langle p-p_{i},n\rangle$ in parameter $w_{2}\left(y\right)$;

(3) On the basis of $\sigma_{1}$ and $\sigma_{2}$, calculate the value of $w_{1}\left(x\right)$ and $w_{2}\left(y\right)$ according to Formulas (12) and (13);

(4) Calculate the bilateral filtering factor, α, and then obtain the new point cloud data after filtering by moving the points in the feature-rich region in the normal direction using Formula (9);

(5) Traverse all the original point clouds to obtain the filtered new point cloud data.

### 2.5. Calculation Method for Phenotypic Traits

In view of current methods for 3D reconstruction of maize plants, the mathematical method was included in this study to describe plant organs, with the aim of reducing data processing and providing objective descriptions of the repeatability and parameterization of growth processes. In this study, the phenotypic traits of plant height, stem diameter and canopy breadth were calculated, which play an important role in evaluating the growth of maize.

#### 2.5.1. Calculation Method for Plant Height

**Figure 4:**
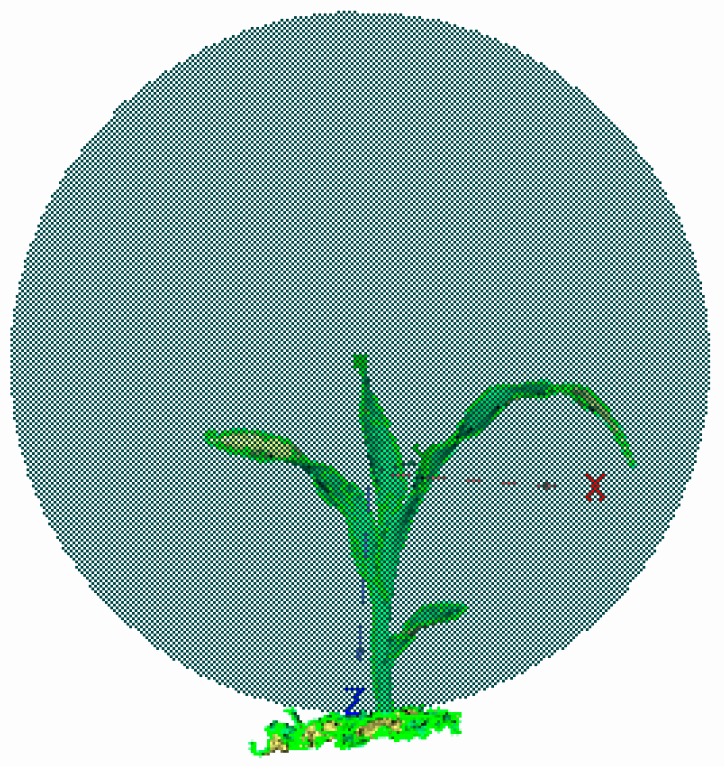
Calculation method for plant height based on the fitting sphere.

Plant height refers to the height difference from the ground to the highest natural extension of leaves. Plant height is an important trait in maize variety cultivation that directly affects the lodging resistance and harvest potential of maize varieties. Therefore, it is of great significance for maize breeding to measure plant height rapidly and accurately. In this study, the y coordinate was taken as the axis, the highest point of the maize canopy in different periods as the vertex, the vertex as the center of the circle, and the lowest point in the vertical direction of the vertex as the base point to form the fitting sphere [43]. The radius of the fitting sphere was the plant height of maize (Figure 4).

To calculate the radius of the fitting sphere and obtain the plant height, the least squares method was used to calculate the plant height of maize. The distance function from any point $P_{i}$ of the fitting sphere to the center of the sphere is as follows:

(16) $$d\left(s,P_{i}\right)=\left|\left|c-P_{i}\right|\right|$$

where $c={\left(S_{1},S_{2},S_{3}\right)}^{T}$ represents the spherical coordinates.

According to the above calculation method for plant height, the distance from any point on the sphere surface to the spherical center was calculated (i.e., the radius of the fitting sphere) as the plant height of maize from the trefoil stage to the jointing stage.

#### 2.5.2. Calculation Method for Stem Diameter

**Figure 5:**
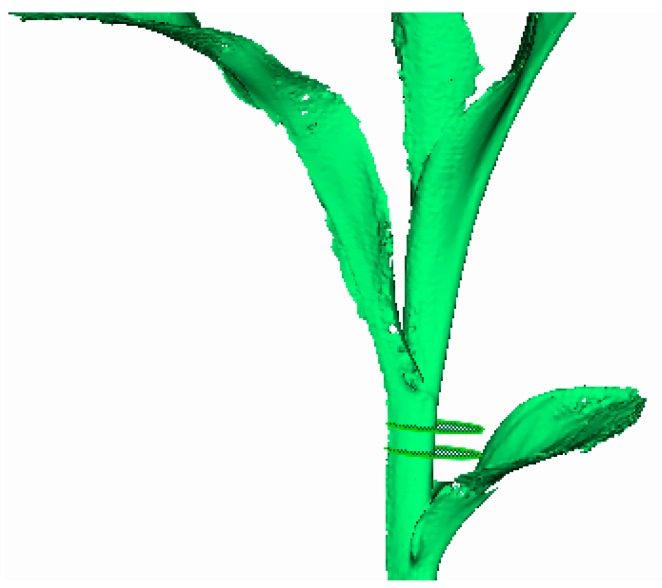
Calculation method for stem diameter based on the fitting cylinder.

Stem growth can indirectly reflect crop growth, vigor, lodging resistance, and so on. Domestic and foreign research has also focused on the measurement of crop stems. For example, the research team at Osnabrück University in Germany designed the high-throughput phenotypic measurement robots Breed Vision [44] and BoniRob [45,46] to measure crop stems and other phenotypic traits by using various optical sensors, such as light curtain imaging, 3D time-of-flight cameras and laser sensors, not only for individual plant phenotyping but also for non-destructive field-based phenotyping in plant breeding. Additionally, Paulus et al. [47,48,49] scanned cereal plants with a hand-held laser scanner and reconstructed 3D models to obtain the stem parameters of crops. Although the above study demonstrated the feasibility of the calculation method for stem parameters involving a hand-held laser scanner, this result was not for stem diameter but for stem height in barley. Stem diameter is a key trait in maize breeding. Thus, to calculate stem diameter accurately, the fitting cylinder was constructed based on a method of coordinate transformation [50]. The diameter of the cylinder was the stem diameter of the maize stem, which consists of nodes and internodes. The diameter of the third internode counted from the bottom to the top was used to indicate the growth status of stems (Figure 5).

The distance equation from any point $P_{i}$ on the surface of the cylinder to the center of the circle is as follows:

(17) $$d\left(s,P_{i}\right)=\sqrt{{\left|\left|q_{0}-p_{i}\right|\right|}^{2}-{\left(a_{0}\left(q_{0}-p_{i}\right)\right)}^{2}}$$

where $q_{0}={\left(S_{1},S_{2},S_{3}\right)}^{T}$ refers to the center coordinate and $a_{0}=\left(S_{1},S_{2},S_{3}\right)$ represents the unit direction vector of the cylindrical axis.

#### 2.5.3. Calculation Method for Canopy Breadth

**Figure 6:**
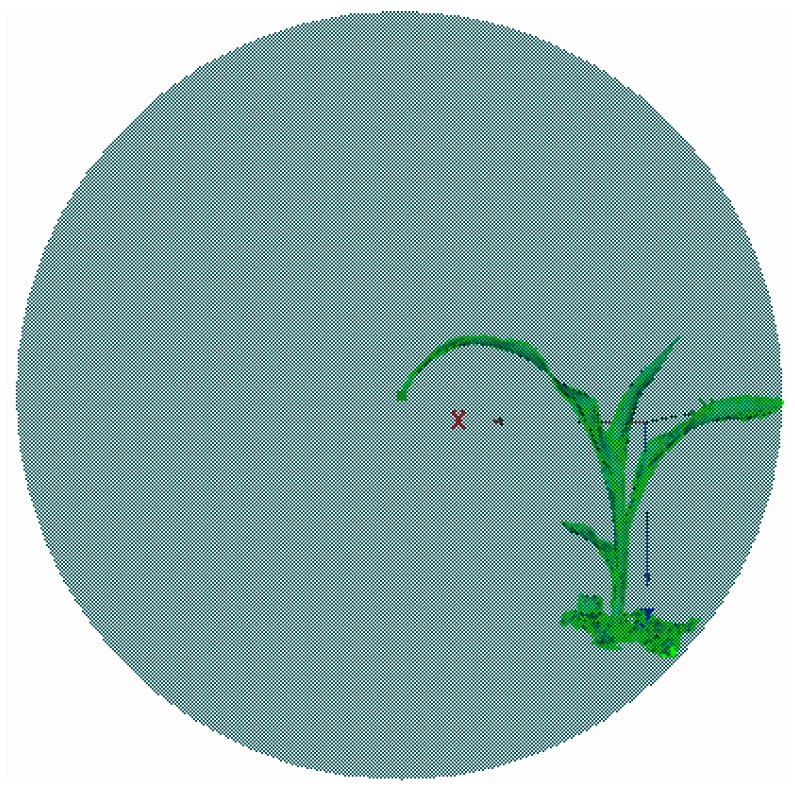
Calculation method for canopy breadth based on the fitting sphere.

Canopy breadth is one of the important reference standards for measuring plant growth. In this study, based on the x-coordinate axis, the left-most point of the maize canopy in different periods was selected as the center of the circle [43]. At the same height as the center, the fitting sphere was generated based on the radius of the distance from the center to the right-most point, and its radius was the plant width of maize (Figure 6). Then, the canopy breadth was obtained with Formula (16).

## 3. Results

### 3.1. Acquisition of Raw Data

When using the FastSCAN 3D scanner to reconstruct the 3D model of the whole maize plant, it was necessary to ensure that the electromagnetic reference body (consisting of a transmitter and a receiver) and the maize plant were in a relatively static state and that neither side could move during the scanning process. To improve scanning quality, the hand-held device (Wand) and the part of maize plant to be scanned were kept perpendicular during the scanning process.

**Figure 7:**
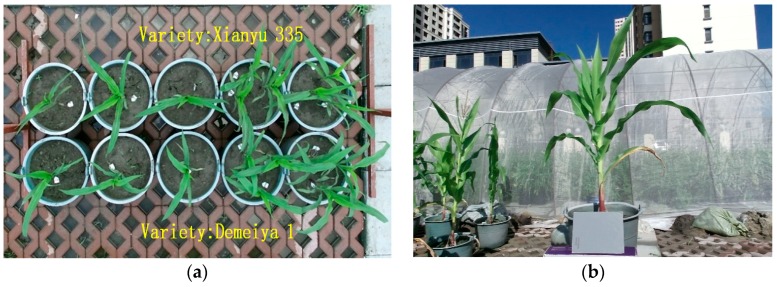
Maize plants under the pot cultivation method. (**a**) A representative sample set of maize plants (**b**) An individual plant.

**Figure 8:**
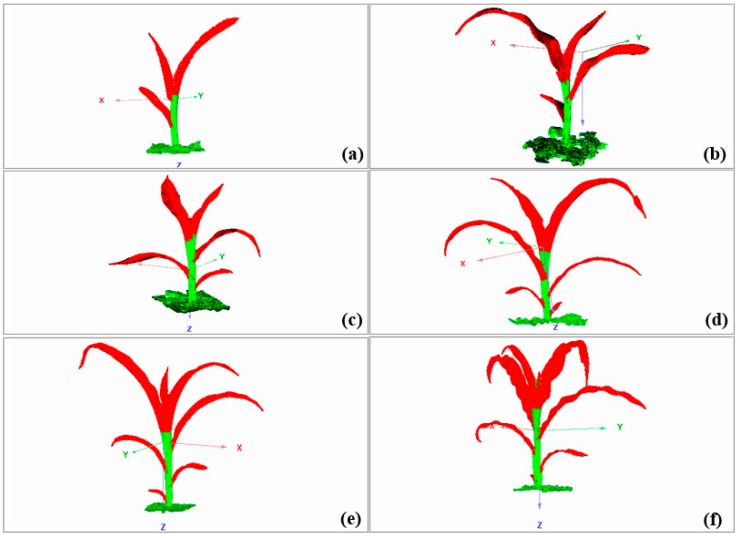
Raw 3D data of maize from the trefoil stage to the jointing stage. (**a**) A maize plant at the trefoil stage; maize plants at the jointing stage, including (**b**) a maize plant with 4 leaves, (**c**) a maize plant with 5 leaves, (**d**) a maize plant with 6 leaves, (**e**) a maize plant with 7 leaves, and (**f**) a maize plant with 8 leaves.

Representative maize plants are shown in Figure 7. The raw 3D point clouds of maize plants from the trefoil stage to the jointing stage were obtained with the FastSCAN 3D scanner, which are shown in Figure 8.

### 3.2. Simplification Effect of Raw Data

To verify the effectiveness of the simplification method, the raw 3D point cloud data of maize plants from the trefoil stage to the jointing stage were simplified. Point cloud simplification was realized on the premise of retaining the details of the 3D model of the maize canopy, and the simplification rate was regarded as the evaluation index, which shown as follows:

(18) $$rate=\frac{P-P_{i}}{P_{i}}\times 100\%$$

where P represents the total number of original 3D point clouds; $P_{i}$ indicates the total number of reduced point clouds; and rate represents the percentage of the total number of simplified 3D point clouds within the raw total.

**Figure 9:**
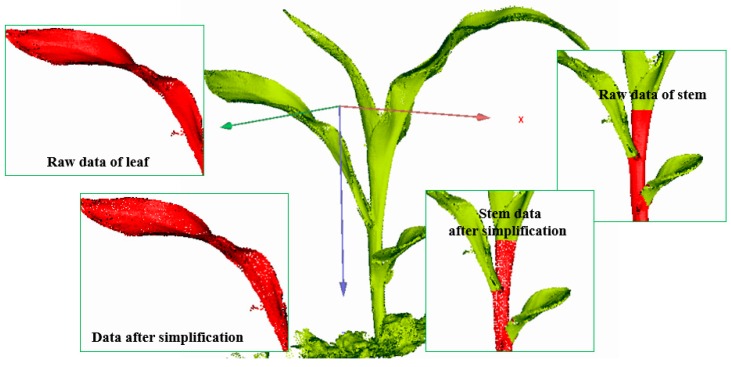
Example of the point cloud simplification effect in maize plants.

Furthermore, the proposed adaptive curvature was applied to simplify the number of points in a flat area, but with the feature details of point clouds being retained in high-curvature areas. According to the height, the raw point clouds were progressively reduced from high to low. Taking randomly selected maize plants as an example, the details of the raw point cloud simplification effect of leaves and stems are illustrated in Figure 9.

The maize canopy, especially the maize leaves and stems, was gradually simplified from the trefoil stage to the jointing stage. The number of point clouds and the reduction rate after the reduction are presented in Table 1.

**Figure 10:**
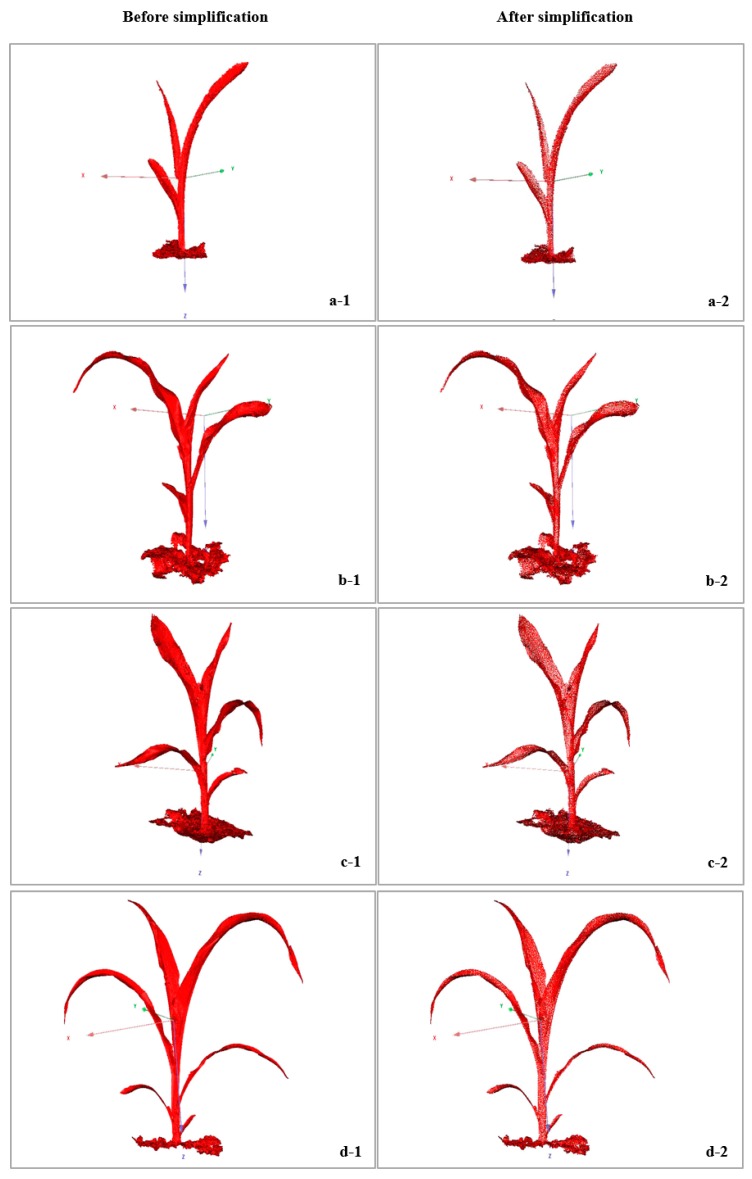

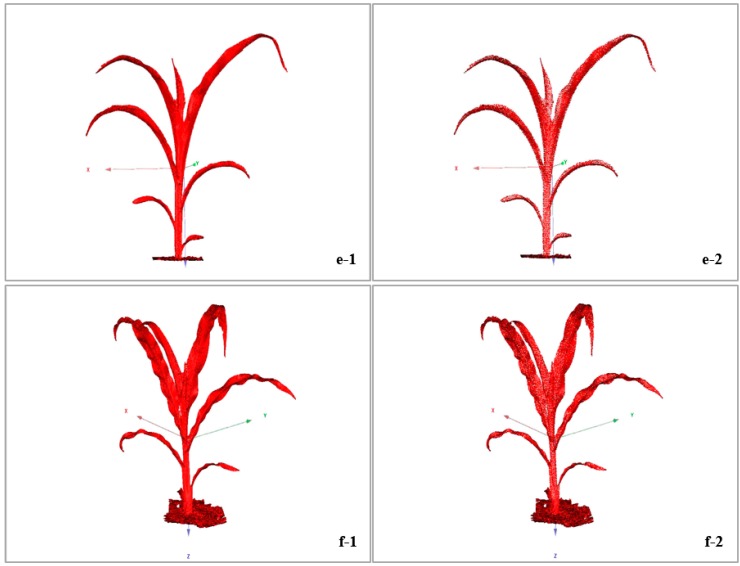
Overall effect after simplification in maize from the trefoil stage to the jointing stage. (**a-1**,**a-2**) A maize plant at the trefoil stage (the number of 3D cloud points was simplified from 10,256 to 7401); maize plants at the jointing stage, including (**b-1**,**b-2**) a maize plant with 4 leaves (the number of 3D cloud points was simplified from 19,587 to 14,521); (**c-1**,**c-2**) a maize plant with 5 leaves (the number of 3D cloud points was simplified from 26,120 to 19,854); (**d-1**,**d-2**) a maize plant with 6 leaves (the number of 3D cloud points was simplified from 30,015 to 23,025); (**e-1**,**e-2**) a maize plant with 7 leaves (the number of 3D cloud points was simplified from 39,542 to 29,525); (**f-1**,**f-2**) a maize plant with 8 leaves (the number of 3D cloud points was simplified from 43,957 to 32,019).

The simplification rate of the raw point cloud in the maize canopy was approximately 25%, which ensured the validity of the simplified point cloud and reduced the processing time of the raw point cloud data. Figure 10 presents the overall simplification effect of the 3D point cloud of maize from the trefoil stage to the jointing stage. Maize plants were simplified via adaptive curvature reduction, and the point cloud model could still retain the morphological characteristics of maize plants. Point cloud simplification was achieved here based on the curvature of the point cloud. The curvature value was positively correlated with the density of the point cloud. Additionally, the degree of point cloud retention was higher in dense areas than in sparse areas.

### 3.3. Denoising Effect of Raw Data

**Figure 11:**
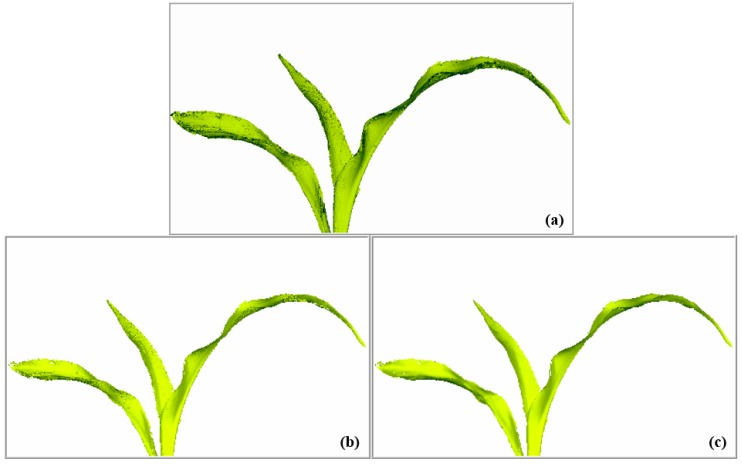
Algorithm comparison for the denoising effect. (**a**) Raw data; (**b**) denoising effect of the Laplace algorithm; (**c**) denoising effect of the bilateral filtering algorithm.

Aiming at an objective evaluation of the denoising algorithm proposed herein, the denoising of the reduced point cloud data of maize plants was processed from the trefoil stage to the jointing stage, followed by comparison of the denoising effect with that of the classical Laplace denoising algorithm. Two objective evaluation indexes, the maximum error and average error, were selected for quantitative analysis of the denoising effect. The comparison results are shown in Table 2. The maximum error was included to measure the maximum distance of point cloud data movement, which was negatively correlated with the quality of the point cloud data. Taking randomly selected maize plants as an example, the denoising effects of some leaves were compared, as shown in Figure 11. The surface of leaves are much smoother using the bilateral filtering algorithm than the effect using the Laplace algorithm, and the edge details of leaves are also preserved well.

**Figure 12:**
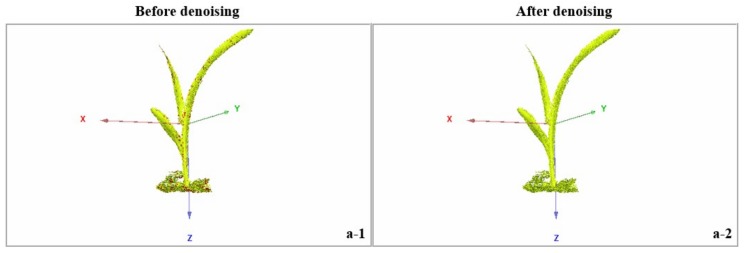

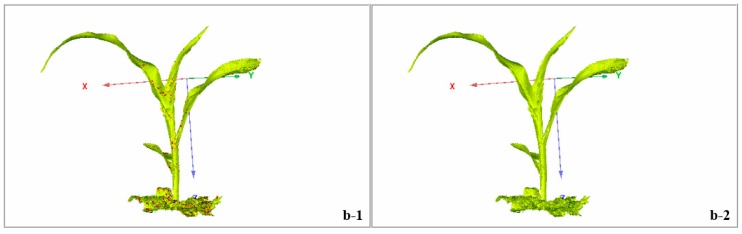

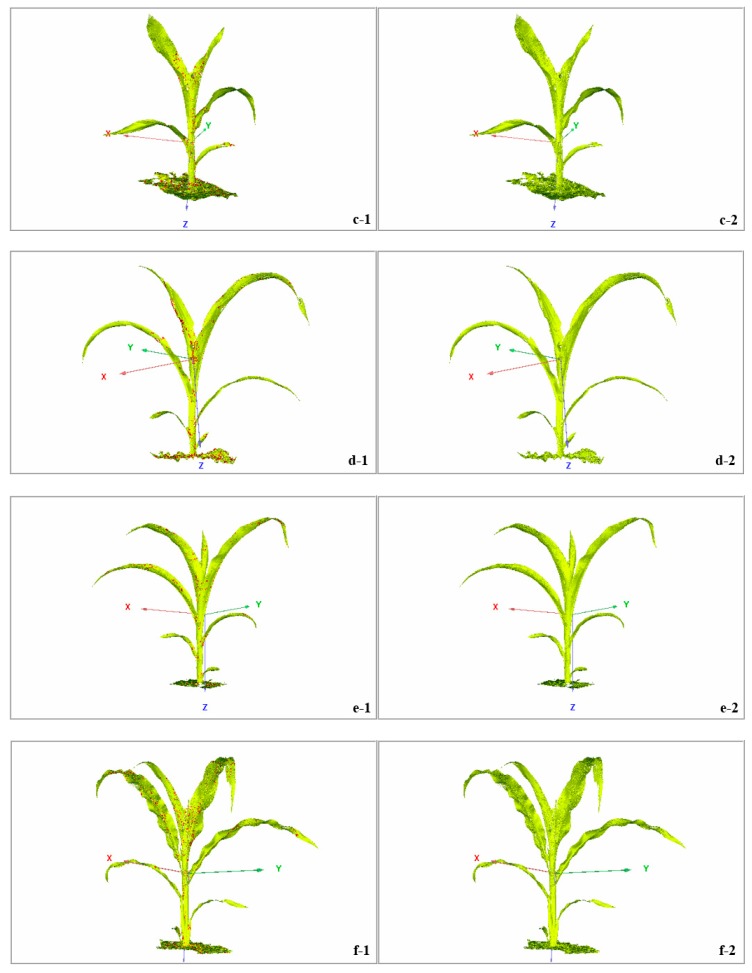
Overall effect of the denoising effect in maize from the trefoil stage to the jointing stage. (**a-1**,**a-2**) A maize plant at the trefoil stage; maize plants at the jointing stage, including (**b-1**,**b-2**) a maize plant with 4 leaves, (**c-1**,**c-2**) a maize plant with 5 leaves, (**d-1**,**d-2**) a maize plant with 6 leaves, (**e-1**,**e-2**) a maize plant with 7 leaves, and (**f-1**,**f-2**) a maize plant with 8 leaves. Red points in the images before denoising indicate the noise points.

The maximum error of the proposed algorithm was 21.1% lower than that of the traditional Laplace filtering algorithm. Additionally, the average error measured the average distance of point cloud data movement, which was positively correlated with the denoising effect of the point cloud. The average error of this algorithm was 38.2% lower than that of the Laplace filtering algorithm. Figure 12 indicates the overall denoising effect of the 3D point cloud of maize from the trefoil stage to the jointing stage. The noise existing in the raw 3D point cloud was labeled with red.

### 3.4. Effectiveness of the Calculation Method for Phenotypic Traits

Ten representative potted maize plants were selected, and phenotypic traits including plant height, stem diameter and canopy breadth were calculated from the trefoil stage to the jointing stage by using the calculation method indicated in Section 2.5, which included 6 periods (3-leaves, 4-leaves, 5-leaves, 6-leaves, 7-leaves and 8-leaves). We calculated and measured the phenotypic traits of 10 potted plants according to each period. Thus, there were a total of 6 periods and 10 samples for each period. The calculated values were compared with the actual measured values.

**Figure 13:**
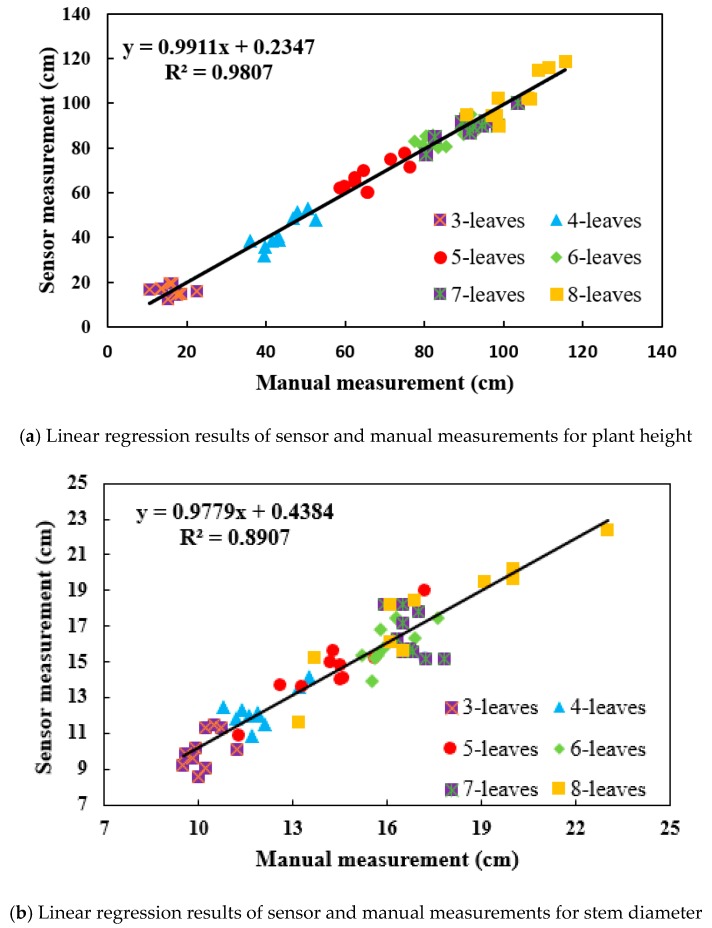

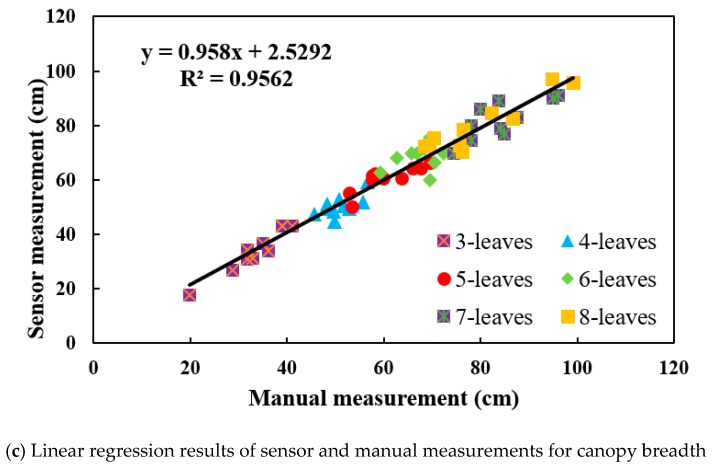
Effectiveness of the calculation method for phenotypic traits.

As indicated in Figure 13a, the calculated value was highly correlated with the measured value based on the calculation method for maize plant height proposed in this study (R^2^ = 0.9807). According to Figure 13b, the method of stem diameter measurement for maize had a better processing effect and could measure the stem diameter more accurately. The determination coefficient, R^2^, of the calculated and measured values was 0.8907. In Figure 13c, the calculated and measured values of canopy breadth are still highly correlated, and the determination coefficient, R^2^, reached 0.9562., which corresponds to the actual phenotypic traits and proves the validity of the proposed method.

## 4. Discussion

### 4.1. Analysis andComparison of the Experimental Results

Manual measurement of the actual phenotypic traits was affected not only by subjective factors related to the surveyor but also by the external environment (e.g., the motion of maize leaves with the wind). Thus, to acquire traits more accurately, actual measurement and data acquisition should be carried out indoors or in a windless environment. In addition, when calculating plant height, stem diameter and canopy width via the method presented in this study, the selection of boundary points [50] in the 3D model of the maize canopy, such as the highest point, lowest point, leftmost point and rightmost point, is also an important factor affecting the calculation accuracy for phenotypic traits.

The calculation accuracy for stem diameter was lower than its counterparts for other traits. In addition to the impact of the external environment and the selection of boundary points, some measurement errors could result from the great interference of the point cloud data at the edge of the maize stem with cylinder fitting. Accordingly, the interference of individual points with cylindrical fitting could be reduced and the accuracy and stability of measurement could be promoted by improving the cylinder fitting algorithm [51,52].

Besides the hand held laser scanner used here, other laser scanning devices, such as a terrestrial laser scanner (Trimble TX8, Danderyd, Sweden) [53], and range cameras like the Kinect v2.0 sensor (Kinect-v2, Microsoft, Redmond, WA, USA) [54] are good selections for phenotying analysis of plants due to their high resolutions, which have been applied in fruit trees and soybean plants respectively in our previous research.

Although data within 120 m can be acquired using a Trimble TX8 3D laser scanner, pre-processing of huge data including lots of redundant data, and registration algorithms of point clouds restrict the speed of 3D reconstruction for plants. Moreover, it takes more time than Trimble TX8 3D laser scanner when acquiring 3D point clouds. For the Trimble TX8, data of at least three stations are needed for reconstructing a maize plant, and it takes at least 5 min to get the data of each station. In order to reconstruct 3D model of a maize plant using data of the three stations, although Trimble realworks software (version: 11.1) [55] can be regarded as a good tool, it will take at least 10 min for reconstruction due to the huge amount of 3D data involved in registration. Nevertheless, it only takes 15 min at most to acquire a 3D model of a maize plant using FastSCAN hand held laser scanner. Thus, the Trimble TX8 laser scanner was not adopted to acquire data of maize plants for comparison with the counterpart of the laser scanner used in this study.

As a new kind of range camera, the red-green-blue-depth (RGB-D) camera consisted of a RGB (red, green, and blue) camera, a depth sensor and infrared emitters has been used extensively in numerous applications [56,57] due to the advantages of low cost and fast speed. Kinect v2.0 sensor is a representative of such cameras and is used extensively in phenotyping analysis of plants [58]. The Kinect v2.0 sensor, originally designed for natural interaction in computer gaming environments, can not only acquire RGB images (1920 by 1080 pixels), but also depth images (512 by 484 pixels) as well as infrared images (512 by 484 pixels) of maize canopies simultaneously with a field of view (FOV) of 70 degrees (H) by 60 degrees (V) and 30 frame rate per second [59]. The Kinect v2.0 sensor was used to acquire 3D point clouds of maize plants and calculate the same phenotypic traits due to its high speed for comparing the accuracy with values calculated using the approach here.

**Figure 14:**
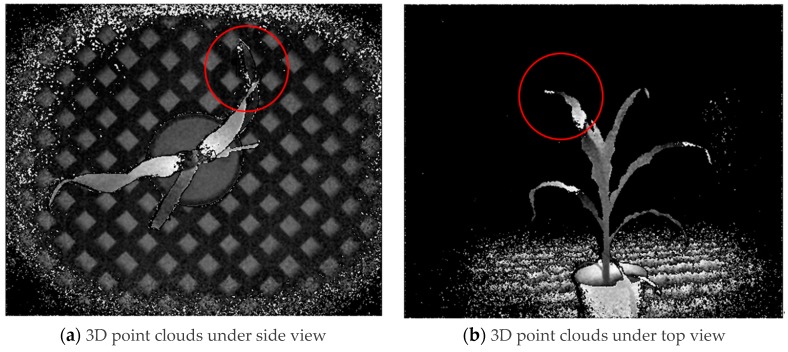
3D point clouds acquired by Kinect v 2.0 sensor.

Two shooting angles were used to acquire 3D point clouds of maize plant (Figure 14). 3D point clouds under top view were prepared for calculation of plant height and canopy breadth, while those under side view were used for calculation of stem diameter.

The determination coefficients (R^2^) were found to be 0.9858, 0.6973 and 0.8854 for plant height, stem diameter and canopy breadth respectively, based on 3D data of the Kinect v 2.0 sensor (Table 3). Compared to the values of phenotypic traits calculated in this study, higher accuracy for plant height was achieved using the Kinect v 2.0 sensor, while R^2^ values for stem diameter and canopy breadth were relatively lower than the counterparts of FastSCAN. Resolution was the first factor impacting on accuracy. With increasing distance, the accuracy of the 3D point cloud decreases from a standard deviation (SD) of a few millimeters to approximately 40 mm, and the point-to-point distance decreases from 2 mm to 4 mm under a FOV of 70 degrees (H) by 60 degrees (V) for the Kinect V2 sensor [59]. In addition, the loss of pixels at the edge of the leaves and stems was another important factor affecting the accuracy, which was worse than that of FastSCAN. Thus, the FastSCAN hand held laser scanner was an excellent device for acquiring the phenotypic traits of maize plants compared to the Kinect v 2.0 sensor in this study.

### 4.2. Advantages and Limitations of the Acquisition System

The present study verified the ability to use a FastSCAN Cobra™ hand-held 3D laser scanner and proposed algorithms for calculating the plant height, stem diameter and canopy breadth of maize plants in an accurate fashion. However, three factors restricted the performance of the proposed approach. First, the FastSCAN Cobra™ hand-held 3D laser scanner is sensitive to strong light, which limits the operation environment of the device. Thus, use of the scanner indoors or under shade outside, without wind, will ensure its effective application. Second, the acquired raw 3D data lack true color information, which is not conducive to research on the color characteristics of a plant canopy. This limitation can be addressed using multi-sensor fusion methods [60,61], such as the combination of 3D point clouds with color information through the registration of a coordinate system between depth sensors and the visible light imaging sensor [62]. Third, the quality of the point cloud and the 3D reconstruction effect is affected by manual scanning operations; redundant points and noise points will increase as the same area is scanned repeatedly, which will occur if the scanning effect of a certain area of a maize plant is not ideal. Although repeated scanning is an unavoidable operation, much improved simplification and denoising methods [63] can be developed to remove the noise of 3D cloud points and improve 3D reconstruction accuracy.

It is important to choose a suitable device for specific phenotyping analysis. Thus, researchers must have an in-depth knowledge of the advantages and limitations of each type of imaging system to accomplish the calculation of phenotypic traits with good accuracy, and efficiency at an affordable cost. The advantages and limitations of common sensors for acquiring geometric traits are shown in Table 4. We finally selected the FastSCAN hand held laser scanner for calculating plant height, stem diameter and canopy breadth according to accuracy and the highest cost performance through comparing their strengths and weaknesses. Although its short measurement range and handheld use have been challenges for the application of FastSCAN in the context of high-throughput phenotype using UAV and other mobile devices such as robotic arms or a vehicle platform, its high resolution and almost perfect 3D modeling effect contributes to its outstanding advantage for calculating fine phenotypic traits, such as stem diameter and leaf inclination angle (LIA) [64].

### 4.3. Future Work

The plant height, stem diameter and canopy breadth of two varieties of maize plants were calculated in this study. From a breeding perspective, a promising approach that should be considered further in future work is the acquisition of other phenotypic traits, such as the leaf inclination angle (LIA) [64], leaf area index (LAI) [65], leaf area (LA) and color indices (CI) [66], to the greatest extent possible. LIA indicates the water stress of plants and impacts on the measurement of LAI [67]. LA refers to an individual leaf, the estimation of LA is important to biometrical observation for a single plant [68]. LAI is not only a ratio of the total area of plant leaves to the land area, but also a comprehensive index of the utilization of light energy within the canopy structure [69]. CI reflects the nutritional status of crops [70]. Additionally, parameters related to the photosynthetic capacity and nutritional status, such as the chlorophyll content and nitrogen content, will be measured during the entire growth stage of maize plants, and the relationship between LIA and LAI [71], CI and nutrition contents will be studied [70].

## 5. Conclusions

In this study, we proposed a method for calculating phenotypic traits based on the 3D reconstruction of maize canopies. The major conclusions are as follows:

(1) An adaptive curvature simplification method of 3D point clouds based on the grid method was proposed. First, the curvature of the marked point cloud was calculated. Second, the size of the outer bounding box was controlled to reduce the 3D point cloud data of maize leaves. The experimental results showed that the whole point cloud was reduced by approximately 25% on the premise of guaranteeing the morphological characteristics of the maize canopy.

(2) Bilateral filtering was used to denoise the feature-rich regions of maize. The maximum error and average error of the proposed algorithm were 21.1% and 38.2% lower, respectively, than those of the traditional Laplace filtering algorithm, providing morphological information for the 3D modeling of maize in a more nuanced manner.

(3) Fitting spheres and cylinders were used to obtain plant height, stem diameter and canopy breadth. The determination coefficients R^2^ were found to be 0.9807, 0.8907 and 0.9562, respectively. The above experimental results suggested that the proposed method for 3D reconstruction of the maize canopy and the approach for calculating phenotypic traits exhibited high accuracy, providing technical support for further study of the phenotypic traits and breeding of other crops.

## Figures and Tables

**Table 1:** Simplification rate of maize from the 3-leaves stage to the jointing stage.

	3-Leaves	4-Leaves	5-Leaves	6-Leaves	7-Leaves	8-Leaves
Raw data	10,256	19,587	26,120	30,015	39,542	43,957
After simplification	7401	14,521	19,854	23,025	29,525	32,019
*rate* (%)	27.8	25.9	24.0	23.3	25.3	27.1

**Table 2:** Comparison of algorithm performance.

	Maximum Error/mm	Average Error/mm
Laplace filtering	19.74	2.54
Bilateral filtering algorithm	15.57	1.57

**Table 3:** Comparison of accuracy for the two devices.

Sensors	Plant Height	Stem Diameter	Canopy Breadth
FastSCAN	R^2^ = 0.9807	R^2^ = 0.8907	R^2^ = 0.9562
Kinect v2.0	R^2^ = 0.9858	R^2^ = 0.6973	R^2^ = 0.8854

**Table 4:** Advantages and limitations of common sensors implemented in acquiring of geometric traits.

Sensors	Distance of Point-to-Point	Advantages	Limitations
Stereo vision system	Various resolutions	Low cost Suitable for unmanned aerial vehicles (UAV)	Heavy computation Sensitive to strong light
Lidar/laser sensor (e.g., Trimble TX8)	7.5 mm at 30 m	Long measurement range High resolution	High cost Limited information on occlusions and shadows
Range camera (e.g., Kinect v 2.0)	>4.0 mm at more than 4 m	Low cost High frame rate	Sensitive to strong light Low resolution
Hand held laser scanner (e.g., FastSCAN)	0.178 mm in the range of 200 mm	High resolution High accuracy for 3D model	Short measurement range Hand held
